# Integrated Molecular Characterization of Intraductal Papillary Mucinous Neoplasms: An NCI Cancer Moonshot Precancer Atlas Pilot Project

**DOI:** 10.1158/2767-9764.CRC-22-0419

**Published:** 2023-10-10

**Authors:** Alexander Semaan, Vincent Bernard, Justin Wong, Yuki Makino, Daniel B. Swartzlander, Kimal I. Rajapakshe, Jaewon J. Lee, Adam Officer, Christian Max Schmidt, Howard H. Wu, Courtney L. Scaife, Kajsa E. Affolter, Daniela Nachmanson, Matthew A. Firpo, Michele Yip-Schneider, Andrew M. Lowy, Olivier Harismendy, Subrata Sen, Anirban Maitra, Yasminka A. Jakubek, Paola A. Guerrero

**Affiliations:** 1 Sheikh Ahmed Center for Pancreatic Cancer Research, The University of Texas MD Anderson Cancer Center, Houston, Texas.; 2 Department of Translational Molecular Pathology, The University of Texas MD Anderson Cancer Center, Houston, Texas.; 3 Department of Radiation Oncology, The University of Texas MD Anderson Cancer Center, Houston, Texas.; 4 Department of Epidemiology, The University of Texas MD Anderson Cancer Center, Houston, Texas.; 5 Department of Surgical Oncology, The University of Texas MD Anderson Cancer Center, Houston, Texas.; 6 Bioinformatics and Systems Biology Graduate Program and Moores Cancer Center, University of California San Diego School of Medicine, San Diego, California.; 7 Department of Surgery, Indiana University School of Medicine, Indianapolis, Indiana.; 8 Department of Pathology and Laboratory Medicine, Indiana University School of Medicine, Indianapolis, Indiana.; 9 Department of Surgery, University of Utah, Salt Lake City, Utah.; 10 Department of Pathology, University of Utah, Salt Lake City, Utah.; 11 Department of Surgery, Division of Surgical Oncology, University of California San Diego, San Diego, California.; 12 Department of Pathology, The University of Texas MD Anderson Cancer Center, Houston, Texas.

## Abstract

Intraductal papillary mucinous neoplasms (IPMN) are cystic precursor lesions to pancreatic ductal adenocarcinoma (PDAC). IPMNs undergo multistep progression from low-grade (LG) to high-grade (HG) dysplasia, culminating in invasive neoplasia. While patterns of IPMN progression have been analyzed using multiregion sequencing for somatic mutations, there is no integrated assessment of molecular events, including copy-number alterations (CNA) and transcriptional changes that accompany IPMN progression. We performed laser capture microdissection on surgically resected IPMNs of varying grades of histologic dysplasia obtained from 23 patients, followed by whole-exome and whole-transcriptome sequencing. Overall, HG IPMNs displayed a significantly greater aneuploidy score than LG lesions, with chromosome 1q amplification being associated with HG progression and with cases that harbored co-occurring PDAC. Furthermore, the combined assessment of single-nucleotide variants (SNV) and CNAs identified both linear and branched evolutionary trajectories, underscoring the heterogeneity in the progression of LG lesions to HG and PDAC. At the transcriptome level, upregulation of MYC-regulated targets and downregulation of transcripts associated with the MHC class I antigen presentation machinery as well as pathways related to glycosylation were a common feature of progression to HG. In addition, the established PDAC transcriptional subtypes (basal-like and classical) were readily apparent within IPMNs. Taken together, this work emphasizes the role of 1q copy-number amplification as a putative biomarker of high-risk IPMNs, underscores the importance of immune evasion even in noninvasive precursor lesions, and reinforces that evolutionary pathways in IPMNs are heterogenous, comprised of both SNV and CNA-driven events.

**Significance:**

Integrated molecular analysis of genomic and transcriptomic alterations in the multistep progression of IPMNs, which are bona fide precursors of pancreatic cancer, identifies features associated with progression of low-risk lesions to high-risk lesions and cancer, which might enable patient stratification and cancer interception strategies.

## Introduction

Pancreatic ductal adenocarcinoma (PDAC) remains a disease with a dismal prognosis and is estimated to become the second leading cause of cancer-related death in the United States within the next decade (1, 2). Although most patients with PDAC present with locally advanced or metastatic disease, a minority of patients are diagnosed with localized disease where curative resection remains an option. Detection and intervention of disease at a localized stage results in a significant survival benefit. PDAC is thought to arise from two distinct subtypes of precursor lesions—approximately 85%–90% of cancers occur on the backdrop of microscopic precursor lesions known as pancreatic intraepithelial neoplasia or PanIN. The remaining approximately 10%–15% are believed to arise from mucinous cystic precursor lesions, of which the vast majority are intraductal papillary mucinous neoplasms (IPMN). Given the projected timeline of several years over which noninvasive precursor lesions progress to invasive neoplasia (3), there is a potentially wide window of opportunity for early detection of this lethal neoplasm.

While PanINs are typically not amenable to noninvasive imaging-based detection, IPMNs have the benefit of being detectable on conventional abdominal imaging studies. Nonetheless, the currently used clinical algorithms, while representing a considerable improvement in stratifying which patients should merit surgery compared with prior schema, continue to both miss incident cancers and overestimate cancer risk in cysts that can be managed conservatively (4). Elucidating the molecular underpinnings of IPMN progression could provide an avenue for identifying those lesions which might be at greatest risk and generate opportunities for early cancer interception.

The genomic landscape of IPMNs has been steadily cataloged over the past decade, and has identified both early drivers that predominate in low-grade (LG) IPMNs, such as *KRAS*, *GNAS,* and *RNF43* mutations, as well as drivers associated with IPMN progression, including *TP53*, *PIK3CA,* and *SMAD4*, among others (5). Recent studies have also revealed genomic heterogeneity within different regions of IPMNs, suggesting most IPMNs originate as polyclonal lesions prior to emergence of a dominant clone (6, 7). Nonetheless, an integrated molecular analysis of IPMNs of varying histologic grades that combines global genomic-wide and transcriptomic analyses has been mostly lacking. This approach can provide unique insights into nongenomic mechanisms of cellular perturbation driving IPMN progression, including potential cross-talk mechanisms with the precursor microenvironment (PME).

Our study was performed as part of the NCI Cancer Moonshot Precancer Atlas Pilot Project (PCAPP), which is a component of the publicly funded NCI Human Tumor Atlas Network (HTAN; ref. 8). In this study, we performed integrated whole-exome (WES) and -transcriptomic sequencing, of LG and high-grade (HG) IPMNs. Our cohort included both independent HG IPMNs (and PDAC), as well as synchronous HG IPMNs arising in the context of a preexisting LG neoplasm. The latter subset is uniquely informative in identifying whether molecular aberrations seen in HG lesions are “wired in” at an earlier stage of dysplasia. We were able to validate many of the “early” and “late” genomic drivers previously reported in IPMN pathogenesis, but also elucidated previously unreported copy-number alterations (CNA) such as chromosome 1q amplification that stratified LG IPMNs at risk of progression to HG IPMNs and PDAC. In addition, our finding reinforces the heterogenous evolutionary trajectories of IPMN progression, which we now demonstrate encompass not only SNVs but also CNAs. At the transcriptomic level, downregulation of transcripts related to antigen presentation was a pervasive feature of IPMN progression, establishing that immune evasion reported in PDAC has its origins in noninvasive lesions.

## Materials and Methods

### Patient Cohort

The HTAN PCAPP in various precursor lesions was organized under the umbrella of the NCI-funded Consortium for Molecular and Cellular Characterization of Screen-Detected Lesions Create (MCL; https://mcl.nci.nih.gov), with MD Anderson leading the PDAC precursor atlas effort. For this PCAPP, 67 histologic samples of IPMN cystic lesions and 10 blood samples from 24 patients, who had informed written consent, were collected between March 2016 and February 2019 across the United States: At the MD Anderson Cancer Center (MDACC) under protocols Lab00-396, PA11-0670, at the Indiana University under protocol number 1011003217 (0209-66), at the University of California San Diego (UCSD) under protocol number IRB_151608 and at the University of Utah under protocol numbers IRB_00011467 and IRB_00089989. Patient 7 [one HG, one Acinar, and one peripheral blood mononuclear cell (PBMC)] was removed for quality issues from DNA analysis for a final of 65 laser-microdissected areas (two PDAC, 22 HG, 17 LG, 15 ND, nine Acinar). All but 2 patients had normal tissue or PBMC for germline correction (nine PBMCs), eight normal duct (ND; laser-microdissected), one normal pancreatic tissue (whole slide), two splenic and one duodenal tissue (both whole slide; Supplementary Table S1). The study was performed in accordance with standard ethical guidelines approved by the Institutional Review Board at every site and in accordance with the Declaration of Helsinki. All patients had clinically and histologically confirmed IPMN. In compliance with the PCAPP guidelines, formalin-fixed paraffin-embedded (FFPE) blocks with a median of 16 months after resection were used (range, 2–33 months). There was no correlation between block age and DNA integrity across institutions which might indicate differences in tissue processing between centers (*R*^2^ = 0.13, *P* = 0.08; Supplementary Fig. S1A).

### Laser Microdissection, Isolation, and Quality Control

All FFPE slides were reviewed by an experienced pancreas/gastrointestinal pathologist at the contributing site and verified at MDACC by one of the authors and an expert pancreas pathologist (A. Maitra). The histologic grade was assigned in accordance with the updated guidelines for preneoplastic precursor lesions in the pancreas (9). Laser microdissection (LCM) and library preparations were centrally performed at MDACC for all samples. Depending on availability, as many as five different areas per patient were collected via LCM using the PALM MicroBeam system (Carl Zeiss Microscopy GmbH) for both DNA and RNA isolation. These area types include ND, acinar cells (AC), LG IPMN and HG IPMN lesions, and PDAC.

An average of three (range, 1–7 slides) consecutive 7 µm, hematoxylin eosin–stained FFPE slides were used for DNA extraction and pooling from each compartment (ND, AC, LG or HG) and a median of three compartments was dissected per patient (range, 1–4). DNA isolation was performed using the QIamp DNA Micro Kit (Qiagen, catalog no. 56304) with a modified protocol: 18 µL of buffer ATL and 12 µL of Proteinase K were combined by vortexing and applied to a customized AdhesiveCap 200 clear (D) (Carl Zeiss Microscopy GmbH, catalog no. 415190-9191-000). Samples were then incubated overnight in an upside-down position at 56°C. A total of 25 µL of buffer ATL and 50 µL of buffer AL were added and pulse-vortexed for 10 seconds. Following, 50 µL of ethanol (100%) were added, pulse-vortexed for 10 seconds and incubated for 5 minutes at room temperature. Lysate was then transferred to QIAamp MinElute columns and centrifuged at 6,000 × *g* for 1 minute. Subsequently, two washing steps at 6,000 × *g* for 1 minute with 500 µL of buffer AW1 and 500 µL of buffer AW2 were followed by a drying step (20,000 × *g* for 3 minutes). Columns were incubated with 20 µL of distilled deionized water for 10 minutes and finally centrifuged at 20,000 × *g* for 1 minute to elute DNA. The DNA obtained from each compartment was then pooled and volume was reduced using the Savant SpeedVac DNA 130 Integrated Vacuum Concentrator System (Thermo Fisher Scientific, catalog no. DNA130-115). Pooled DNA concentration was measured using the Qubit dsDNA BR Assay Kit (catalog no.: Q32853, Qubit 2.0 fluorometer). DNA was stored at −20°C until further processing.

In addition, bulk germline DNA (gDNA) was extracted from two 7 µm FFPE using the QIAamp DNA FFPE Tissue Kit (Qiagen, catalog no. 56404). gDNA integrity was measured by the genomic DNA ScreenTape (Agilent, catalog no. 5067-5365) on a Tapestation 2200 system in conjunction with TapeStation Analysis Software (Agilent). Median DNA integrity number was 4.95 (range, 2.4–6.2). Matching whole blood samples were collected in acid citrate dextrose tubes (BD) and processed within 3–4 hours of phlebotomy (*n* = 10) as described previously (10). Whole blood was centrifuged at 2,500 × *g* for 10 minutes to separate plasma. PBMCs were isolated using the Lymphocyte Separation Medium (Corning, catalog no. 25-072-CV) and centrifugation at 620 × *g* for 30 minutes. PBMC DNA was isolated using the DNeasy Blood & Tissue Kit (Qiagen, catalog no. 69506) following the manufacturer's protocol.

### DNA Library Construction and Sequencing

A median of 63 ng (range, 10–200 ng) for FFPE derived, pooled DNA and a median of 155 ng (range, 105–200 ng) of matched PBMC DNA was fragmented using the SureSelect XT HS and XT Low Input Enzymatic Fragmentation Kit following the manufacturer's instructions (Agilent, catalog no. 5191-4080). Molecular-barcoded libraries were constructed following the SureSelect XT HT targeted enrichment protocol for Illumina paired-end multiplexed sequencing libraries (Version A1, July 2017) as described previously (10) with the following modifications: Step 2.4: Incubation at 20°C for ligation for 35 minutes, step 3.1: Precapture pooling of samples were used if necessary to reach a minimum input for the hybridization step of 50 ng and incubation temperature for segment numbers 2–5 were reduced to 62.5°C. SureSelect Clinical Research Exome V2 (Agilent, catalog no. 5190-9492) and Exome V7 (Agilent, catalog no. 5191-4005) was used for capturing. For cross-validation samples (*n* = 6), the All-In-One solid tumor panel (AIO, Agilent, catalog no. G9706S) was used for capture. Final libraries were multiplexed, denatured, and diluted to a final concentration of 1.7 pmol/L for sequencing and cluster generation as per manufacturer's recommendation. Clustered flow cells were sequenced on the Illumina NextSeq 500 instrument targeting 400× coverage (Illumina) using standard Illumina paired-end primers and chemistry (index 1 = 8, index 2 = 10, read length = 125).

### Analysis of Mutations and CNAs

#### Alignment and Processing

Raw sequencing data were converted to fastqs with bcl2fastq (v2.20.0.422), including a molecular barcode fastq. Fastq files were assessed for quality with FastQC (v0.11.8), trimmed with SureCall Trimmer (AGeNT, Agilent, v4.0.1) to remove adaptor sequences, and then aligned to hg19 with Burrows-Wheeler Aligner (0.7.15-r1140). The resulting BAM was then collapsed by barcodes to family size of one, according to default parameters for LocatIt (AGeNT, Agilent, v4.0.1) with two exceptions: without filtering for barcode quality (*q* = 0), and correcting for optical duplicate detection (*c* = 2,500) to account for sequencing on patterned flow cells. Collapsed bams were then processed for base quality score recalibration according to best practices by the genome analysis toolkit (GATK, 4.1.2.0), using dbSNP138 to exclude consideration. Samples that failed quality control (QC) were resequenced.

#### Mutation Calling

Three callers were used for somatic variant detection: Mutect2 (GATK, v4.1.3.0), SureCall (Agilent, 4.1.1.9), and MuSE (v1.0rc). Across all three callers, tumor samples were run against a paired normal based on availability (order priority: PBMC, uninvolved non-pancreatic tissue, NDs). Mutect2 was run according to best practices and default parameters, including checking for cross-sample contamination, and filtering for sequencing artifacts (such as orientation-based FFPE artifacts). To further reduce possible population variation, a panel of normal DNA with all available PBMCs and NDs (without detectable *KRAS* and *GNAS* mutations) was used as an additional filter. When no paired normal was available, Mutect2 was run in tumor-only mode, filtering against the panel of pooled normal sequences. A set of high-confidence Mutect2 calls was generated by further filtering with a CONTQ score of 50 or greater. SureCall was run from BAMs according to the Agilent-provided defaults: “Default SureSelect Tumor Normal Method” when paired data were available, or by “Default SureSelect” method in single sample mode when not. MuSE was also run according to default parameters, and variants meeting the “PASS” or “Tier1” criteria were included in downstream analyses. Because matched normals are required for MuSE, two samples (5 and 19) did not have MuSE calls. Finally, VCFs were annotated by ANNOVAR (2018-04-16) across a variety of metrics, including gnomAD, ExAC [non-TCGA (The Cancer Genome Atlas)], COSMIC86, and ClinVar. SNVs called by two or more of the callers were used for downstream analysis. When MuSE calls were unavailable, the union of Mutect2 and SureCall SNV calls was used for downstream analysis. All SNVs with an allele frequency greater than 1% in gnomAD or ExAC (non-TCGA), were removed.

The SNV call set from this agnostic approach was complemented by a set of variants identified through a targeted analysis at predetermined genomic locations identified by only one caller and requiring less stringent filtering parameters. These predetermined genomic loci included two groups, those in established PDAC driver genes and SNVs detected by the more stringent approach in other samples from the same patient. These included a set of mutect2 variant calls that were generated under more sensitive conditions (lowering the log odds threshold for emission to 1.5). PDAC driver genomic loci were defined as those with 15 or more entries in the COSMIC database in established PDAC genes (refs. 11, 12; *KRAS; TP53; SMAD4; CDKN2A; GNAS; BRAF; PIK3CA; MAP2K4; TGFBR1; TGFBR2; RNF43; CTNNB1; STK11; ARID1A; KDM6A; SF3B1; RBM10; IDH1; PTEN; APC; ATM; BRCA1; BRCA2*). In addition, SNVs were called using this targeted approach at genomic sites where an SNV had been detected using the two-caller approach in at least one of the patient's other nonblood samples.

SNVs were classified as deleterious if they were an exonic or splicing variant, and if they were labeled as deleterious by two or more prediction models (sift, polyphen, HVAR, LRT, mutationTaster, fathmm, provean), or if the variant was labeled as pathogenic in ClinVar. Small insertion and deletion calls were generated using Pindel (0.2.5b9) with the default parameters and filtered by depth (minimum 5 supporting reads in tumor, maximum 0 corresponding reads in normal).

Analysis of mutation burden and detection of CNAs was performed as described previously (10). Briefly, two independent algorithms were used for detection and classification of CNAs. HapLOHseq (13) was used for detection of genomic regions exhibiting allelic imbalance (AI) with results from this algorithm being combined with output from standard log_2_ copy ratio segmentation data. GATK was used for segmentation of log_2_ copy ratio data (14). CNAs were called by overlaying HapLOHseq AI and GATK segmentation calls. Clonal lineages were inferred using the Metastatic And Clonal History INtegrative Analysis (MACHINA), an algorithm that models the evolutionary trajectory and migration histories of clones in metastatic cancer using SNV data (15). We categorized the SNV generated phylogenetic trees for each patient as “linear” or “branched”. Linear evolution was defined when a clone in the LG acquired mutation(s) in a stepwise manner to give rise to a dominant clone present in the HG lesion. A branched evolution is defined by branching of the HG and LG lineages leading to the dominant HG clone being found an independent branch that is not shared with the LG.

To ensure high-quality SNV calling, we performed cross-validation including parallel digital droplet PCR (ddPCR) mutation calling for *KRAS* and *GNAS* in all samples and parallel, ultra-deep targeted panel sequencing. Both methods showed a high concordance of the called mutations (ddPCR: *R*^2^ = 0,95, *P* < 0.0001; Supplementary Table S2; Supplementary Fig. S1B).

### TCGA 1q Analysis

Previously published TCGA PDAC data were analyzed to determine an association between the RNA molecular subtype and 1q whole arm amplification. RNA-based classifications (Moffitt, Collissson, and Bailey) and copy-number calls were obtained from previous publications (11, 16). This analysis was limited to TCGA PDAC samples with a consensus classification of basal or classical for all three classifiers. A *χ*^2^ test was used to determine significance (18 classical with 1q gain, 16 classical without 1q gain, three basal with 1q gain, and 15 basal without 1q gain; *P* = 0.017).

### RNA Isolation, Library Construction, and Sequencing

For RNA, tissues were harvested directly into caps by LCM as described above, collecting 500–1,000 cells as input per library, and stored at −80°C until ready for use. We processed the tissue via a modified SMART3 protocol (14, 17). Briefly, we performed steps as described by Foley and colleagues up to PCR. PCR was performed as described except for cycle number (20, 21, or 22) was additionally dependent on planned post-PCR replicate pooling (3, 2, or 1 respectively). Following PCR amplification, libraries from the same patient and tissue were pooled, cleaned up using beads, and stored in RNase-free water. Samples were indexed with indices 1–16, 18–20, 22, 25, and 27 from ref. 14 corresponding to TruSeq LT indices. Prepared libraries were quantified and checked for appropriate size distribution using an RNA ScreenTape (Agilent, catalog no. 5067-5579) on a Tapestation 2200 system in conjunction with TapeStation Analysis Software (Agilent), and stored at −80°C until ready for sequencing. When ready, libraries were pooled, multiplexed, and diluted to a final concentration of 1.6 pmol/L for sequencing on a NextSeq 500 (Illumina), single-end, with 75 cycles (Illumina, catalog no. 20024906).

Raw files were processed as described previously (17). Briefly, raw data from the sequencer were converted to fastqs with bcl2fastq (v2.20.0.422) and fastqs were assessed for quality with FastQC (v0.11.8). The 3SEQtools suite (https://github.com/jwfoley/3SEQtools) was used to further process the data including read trimming for adaptor contamination and polyA tails (FastQC), alignment with STAR (2.7.1a), and depth-aware deduplication (3SEQtools). Finalized bams were processed for differential expression using DESeq2.

To infer PDAC molecular subtypes in our samples, we used an approach previously utilized in hepatocellular carcinonoma (18). SCnorm was used to normalize expression as it has been used for single-cell RNA sequencing (RNA-seq; ref. 19). Subtype classification was done using nearest template prediction and the package CMScaller was used as a wrapper for the nearest template prediction function. Finally, we applied the Moffitt (20), Collison (21), and Bailey (22) classifiers to each RNA-seq replicate.

### ddPCR Analysis

ddPCR was performed using whole-slide FFPE-extracted DNA on a QX200 Droplet Digital PCR System (Bio-Rad) following previously described protocols (23, 24). For highly sensitive multiplex *KRAS* and *GNAS* detection, we used specific *KRAS* probes following the manufacturer's protocol (Bio-Rad) for G12V (catalog no. dHsaMDV2510592), G12D (catalog no. dHsaMDV2510596), G12R (catalog no. dHsaCP2506874), GNAS probes (Bio-Rad), R201S (catalog no. dHsaMDS2513808), R201C (catalog no. dHsaMDV2510562), and R201H (catalog no. dHsaMDV2516796).

### Neoepitope Prediction

Personalized HLA types were generated from the WES data using OptiType (1.3.1) and confirmed using PolySolver (1.0). For Optitype, finalized DNA bams were deconstructed back into paired-end fastqs with BEDtools (bamtofastq, 2.28.0). Each paired-end fastq was then processed separately and aligned to an HLA reference with RazerS3 (3.5.7). The resulting BAMs are then deconstructed a second time with SAMTOOLS (bam2fq, 1.9), to create fished/filtered fastqs. These fastqs are then processed together with OptiType with default parameters to generate an HLA type for each person/tissue. For PolySolver, the finalized bams are processed directly. HLA types are considered confirmed when both OptiType and PolySolver agree.

Personalized variant antigens by cancer sequencing (pVACseq), from the cancer immunotherapy tools suite, pVACtools (v1.5.2) was then used for neoepitope prediction. VCFs from both the mutect2 and MuSE pipelines were filtered by the list of consensus calls, annotated with Variant Effect Predictor (VEP, v94), to which coverage was also added using kallisto (0.44.0). We also added expressions of the transcript according to the SMART3 data with the IPython package VAtools (vcf-readcount-annotator) and kallisto. Pindel calls were restricted to established cancer genes (25) and processed similarly. The VEP-, coverage- and expression-annotated VCFs are then processed with pVACseq, using the confirmed personalized HLA types, an epitope size ranging from 8 to 11, and with multiple algorithms specified (MHCflurry, MHCnuggetsI, NetMHC, NetMHCpan, PickPocket, SMM, and SMMPMBEC). The resultant list of predicted neoepitopes was then combined per patient/tissue, checked for duplicates (e.g., variants called in both Mutect2 and MuSE).

### Statistical Analysis

Statistical analyses were performed with Prism 8 (Graph Sotfware, Inc.) and statistical significance was determined as a *P* value of <0.05.

### Data Availability

All datasets generated in this study have been uploaded into database of Genotypes and Phenotypes (dbGaP) under accession number phs002225.v3.p1.

## Results

### IPMN Cohort and Clinicopathologic Features

A total of 67 histologic samples from 24 surgically resected IPMN cases were analyzed for their genome and transcriptome expression. Two samples (one HG and one acinar) failed initial QC control and were excluded from further analysis, leaving 65 laser-microdissected histologic areas for assessment (Supplementary Table S1). Two of the processed FFPE blocks for analyses contained invasive carcinoma, although 9 of 23 of the final pathologic reports indicated an invasive carcinoma (median maximum diameter of 6 mm, range <1–50 mm). Patient data and clinical annotations are summarized in Table 1 and an experimental outline can be found in Supplementary Fig. S1C and S1D.

**TABLE 1:** Patient demographics and clinicopathologic characteristics

Cohorts characteristics	Patients
Total patient number	24
Peripheral blood samples	10
Non-tumorous tissue sample	9 pancreatic tissue, 1 duodenum, 2 spleen
Age of resection (median)	70 years (range, 56–82 years)
Gender	
Men	15 (62.5%)
Women	9 (37.5%)
Maximum diameter IPMN (median)	27.5 mm (range, 7–50)
Carcinoma detected	9 (37.5%)
Histologic grading	
Intermediate	9 (37.5%)
High	15 (62.5%)
Histologic subtypes IPMN	
Intestinal	8 (33.3%)
Pancreatobiliary	12 (50%)
Gastric	4 (16.6%)
Relation to main duct	
Side branch	6 (25%)
Main duct	6 (25%)
Main and side branch	3 (12.5%)
Not reported	9 (37.5%)
Cyst location	
Head	13 (54.1%)
Tail/Body	10 (41.7%)
Diffused	1 (4.2%)

### *KRAS* and *GNAS* Mutations in IPMNs

**FIGURE 1:**
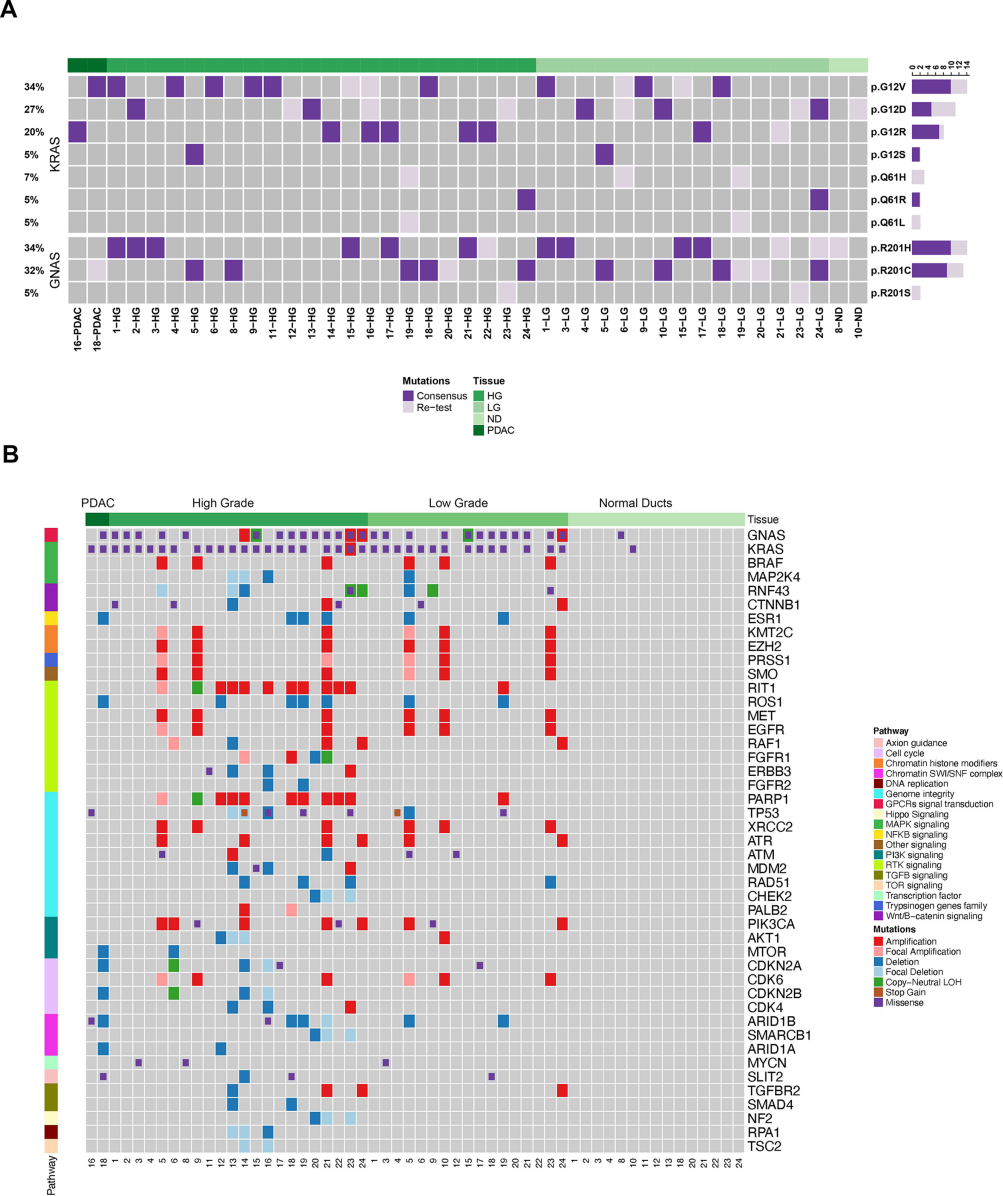
Genomic landscapes of IPMN lesions. **A,** SNVs identified in *KRAS* and *GNAS* in LG and HG IPMNs. Dark purple: somatic mutations detected for *KRAS* and *GNAS*. Light purple: mutations detected by retesting. **B,** SNVs and CNAs identified in ND, LG, HG, and PDAC regions and classified by relevant PDAC-related pathways. For A and B, samples were arranged by histological type and labeled at the top of each heat map.

Our WES approach indicated that most premalignant lesions either harbored *GNAS* or *KRAS* mutations (65.2% and 91.3%, respectively). In 9 patients, the synchronous LG and HG samples demonstrated the same *KRAS* and *GNAS* mutations. Furthermore, 2 patients harbored the same *KRAS* mutations in synchronous LG and HG samples, while 2 additional patients had the same *GNAS* mutations in synchronous LG and HG samples (Fig. 1A). These data reinforce the well-established paradigm that *KRAS* and *GNAS* mutations are “early” genetic drivers acquired prior to progression. Moreover, in 4 patients, we detected multiple *KRAS* mutations, either in the LG (patient IDs 6, 19, and 24) or in HG (patient ID 16), reiterating the prior observation of independent clonal events in IPMN pathogenesis (26), especially in LG lesions, with convergent evolution upon subsequent progression. Finally, in patient 10, we documented a shared *KRAS* mutation in the LG and matched ND, although this was the only ND sample to harbor a *KRAS* mutation.

### Genetic Alterations in Premalignant Lesions

On average, 34 nonsynonymous mutations were detected per microdissected LG and HG region with a mean mutation burden (MB) of 0.91 mutations per megabase (Mb) which is consistent with previous reports [41 mutations and 1.11 MB, (19)]. We compared the mutational load from LG with HG lesions and found no differences in the number of mutations by an unpaired and paired analysis (Supplementary Fig. S2A and S2B). We analyzed CNA events in precancer lesions and classified alterations as focal when smaller than 3 Mb (average 0.8 Mb and median 0.65 Mb). Those CNAs not classified as focal had an average size of 17 Mb (median 8 Mb). An overview of all CNA events per patient is shown in Supplementary Fig. S2C. Mutations and CNAs were identified in genes belonging to pathways such as MAPK, RTK, and TGFβ signaling, as well as genome instability and cell cycle (Fig. 1B). Among others, these CNAs involved genes in RTK signaling (*RIT1, ROS1, MET, EGFR, RAF1, FGFR, ERBB3, FGFR2*), genome integrity (*TP53, PARP1, XRCC2, ATR, ATM, MDM2, RAD51, CHEK2, PALB2*) and cell cycle (*CDKN2A, CDK6, CDKN2B, CDK4*; Fig. 1B; Supplementary Fig. S2C).

**FIGURE 2:**
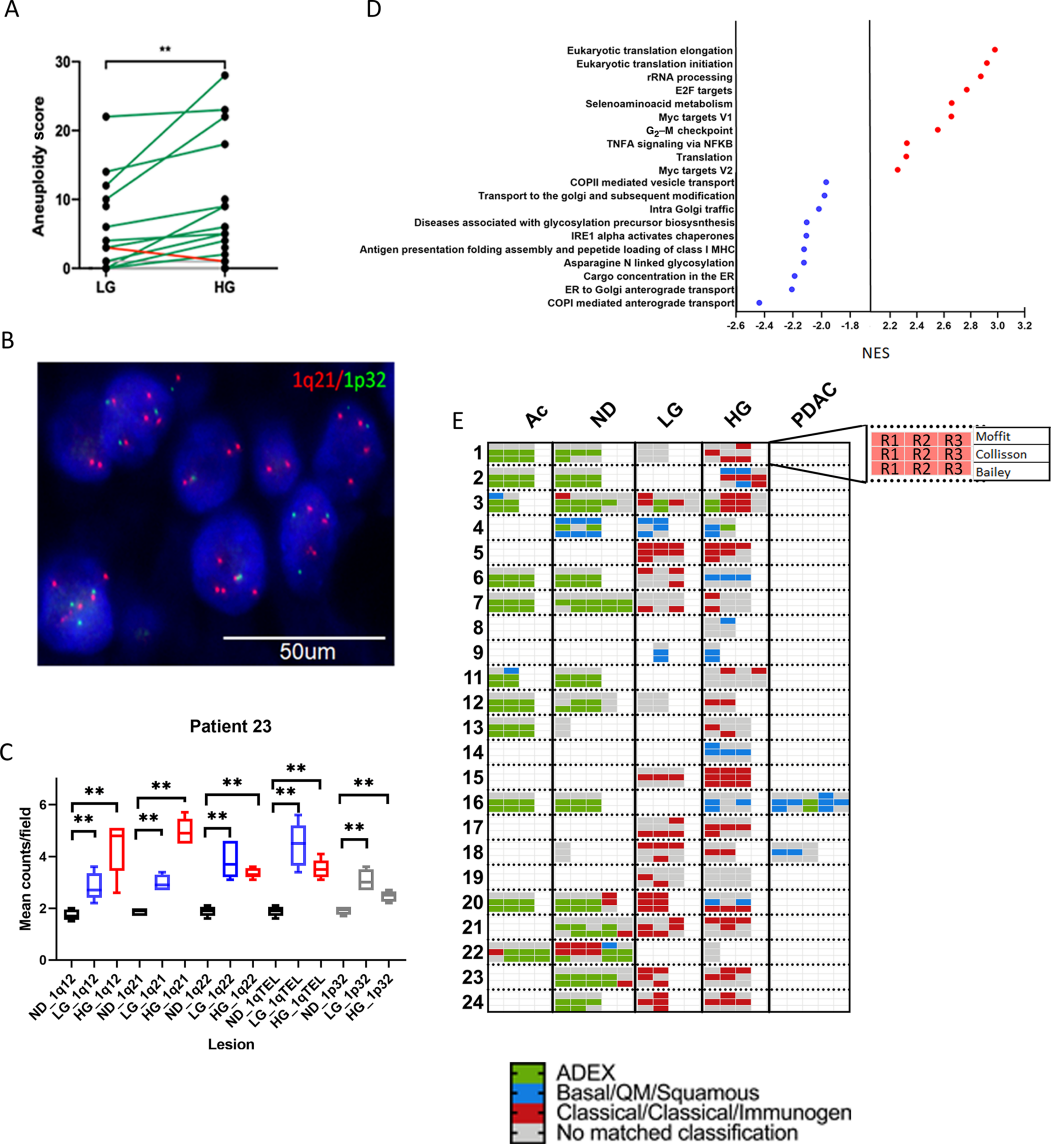
Comparison of CNAs between LG and HG IPMNs. **A,** AS between LG and HG by paired analysis, green lines show an increase of AS upon progression while red lines a decrease. Validation (**B**) and quantification (**C**) of chromosome 1q amplification by FISH (ND: black colored bars, LG: blue colored bars, HG: red colored bars, gray reference 1p results). **D,** NES results for pathways upregulated and downregulated in HG versus LG (FDR < 0.01, *P* = 0), red and blue color highlights important pathway in PDAC. **E,** Expression of PDAC molecular subtypes in precancer cystic lesions per patient; 2–5 replicates (single colored box) were sequenced per region with each row representing classification by Moffit (top), Collisson (middle), and Bailey (bottom) and organized as it is shown in the inset.

We then calculated an aneuploidy score (AS) for each sample as described previously, defined as the number of chromosome arms spanned by a CNA (minimum 75% of the chromosome arm; ref. 27). The AS was significantly higher in HG regions compared with LG regions (Fig. 2A; Supplementary Fig. S2D). One of the most frequent alterations was amplification of the 1q arm (Supplementary Fig. S2C and S2E; 9 patients, 10 HG or LG regions). We performed FISH as an orthogonal validation method. Probes expanding locations 1q12, 1q21, 1q22, 1q telomere, and 1p32 (control) were utilized in two cases containing paired LG and HG lesions (IDs 18 and 23); all q probes showed a significantly higher mean count field foci compared with normal ducts (Fig. 2B and C). In addition, 1q amplification was more common in HG (9/23) versus LG (1/17; Supplementary Fig. S2E) and the coding regions for *PARP1* and Ras-like without CAAX 1 (*RIT1*) were located within the amplified loci. Therefore, we performed integrated analysis by RNA-seq and found significant overexpression of transcripts for both genes in lesions with gains in 1q compared with unaltered regions (Supplementary Fig. S2F and S2G). Furthermore, all IPMN lesions that showed 1q amplification showed a higher AS (*P* < 0.0001) but not higher TMB (*P* > 0.05; Supplementary Fig. S2H and S2I). In this regard, *PARP1* plays a critical role in the DNA damage repair, including highly error-prone DNA repair that enhances genomic instability (28, 29). In addition to *PARP1*, we also investigated the presence of genetic alterations in other chromosomal instability–related genes. 22 out of 39 preneoplastic lesions harbor SNVs and/or CNAs in *PARP1, TP53, XRCC2, ATR, ATM, MDM2, RAD51, CHEK2,* and *PALB2* which correlated with significantly higher AS in these lesions (Supplementary Fig. S2J). In summary, CNAs appear to be common event in noninvasive IPMNs, increase upon progression to HG lesions, and specifically, 1q amplification appears to stratify for IPMNs at higher risk of progression.

We compared the evolutionary trajectory between cases with co-occurring carcinoma and those that did not progress to PDAC. In addition to the two PDAC cases at hand (patient IDs 16 and 18), there were seven additional samples with coexisting carcinoma (patient IDs 8, 9, 12, 14, 20, 21, and 22) in the final pathology report. At the DNA level, these carcinoma co-occurring IPMN showed no differences in either MB or AS between LG or HG. Importantly, seven of nine samples with coexisting carcinoma showed chromosomal aberrations in chromosome 1q (Supplementary Fig. S2C) and eight of nine contained mutations in chromosomal instability–related genes. This suggests the potential significance of a chromosome arm 1q amplification as a progression marker in IPMN.

### Transcriptomic Analysis of IPMNs

To evaluate transcriptomic signatures driving the LG to HG progression, we analyzed bulk RNA-seq data from microdissected LG and HG lesions that were processed by the Smart-3SEQ approach (17). Differential gene expression followed by gene set enrichment analysis between HG and LG lesions indicated that progression to HG was associated with downregulation in pathways related to antigen presentation and glycosylation compared with LG lesions (FDR < 0.01, *P* value = 0; Fig. 2D). In contrast, HG lesions were enriched for pathways more closely related with PDAC biology such as oncogenic MYC targets [normalized enrichment scale (NES): 2.657, FDR: 0.0], E2F targets (NES: 2.772, FDR: 0.0), cell cycle, and translation (Fig. 2D). In addition, we characterized the expression of potential neoepitopes and their presentation. We applied WES data to OptiType (30) and PolySolver (31) to perform neoepitope prediction and identified 20 genes with at least 10 potential neoepitopes which were significantly enriched in HG compared with LG (Supplementary Fig. S2K and S2L). Despite the putative higher neoantigen load, the downregulation of transcripts associated with the antigen presentation machinery in HG lesions suggests that immune evasion in cancer has its origins within the PME of noninvasive precursor lesions.

### Transcriptomic Subtype Classification

Next, we studied whether well-established transcriptomics subtypes of PDAC (basal-like and classical) could be identified in noninvasive IPMN. Using commonly accepted gene sets (32), majority of all regions analyzed were classified into the two subtypes (Fig. 2E; Supplementary Fig. S2M). While in some IPMN samples, more than one subtype was detected (e.g., Patient ID 2 and 20), in others, a class switch was found upon progression (Patient ID 6, 18, and 20; Fig. 2E). For example, the IPMN lesion in patient 20 underwent a subtype class switch (classical to basal-like) upon progression from LG to HG. Of note, ACs and normal ducts were almost exclusively classified as ADEX/Exocrine, reflecting that this previously described subtype might reflect normal tissue contamination (ref. 33; Fig. 2E). Interestingly, the majority of HG lesions with 1q amplification (6/9) were classified as classical subtype (Fig. 2E). We further validated this observation through TCGA cohorts which demonstrated an association of the classical subtype with 1q gains (*P* = 0.017; Supplementary Table S3).There was no difference between classical and basal assignment of IPMN lesions with regards to overall genomic characteristics like MB or markers of genomic instability like aneuploidy. Nonetheless, mutations in *GNAS* were predominantly seen in classical subtypes (Supplementary Fig. S2N).

### Evolutionary Trajectory in Precancer Lesions: SNVs versus CNAs

Previous work has demonstrated a highly heterogeneous progression pattern of IPMNs by SNVs (6, 7, 26, 34), which is confirmed in our cohort. Clonal evolution was evaluated by two different approaches (see Materials and Methods for details). SNV analysis indicated that 11 of 23 (48%, ID cases 1, 3–5, 9, 15, 17–19, 21, and 23) cases showed a linear evolution, while in the minority of cases, five of 23 (22%, ID cases 6, 12, 20, 22, and 24), the HG lesions showed a branched evolution from LG. In addition, in seven of 23 (30%) the evolution could not be inferred. We then compared the molecular subtypes derived from RNA-seq in lesions with their evolutionary trajectory based on SNV calls. In patients with linear evolution, the molecular subtype persists in most cases (9/11) during transition from LG to HG, while in patients with branched evolution, the molecular subtype of the LG IPMN is present in only one of five matched HG lesions.

**FIGURE 3:**
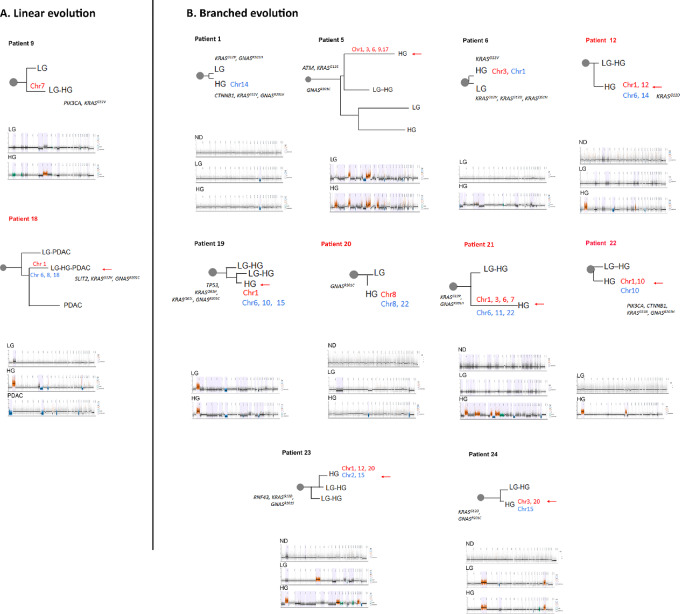
Inferred evolutionary trajectory derived from CNAs. Cases showing linear (**A**) and branched (**B**) evolution are depicted. For each case, evolutionary tree and segmentation plots are shown with HapLOHseq calls represented in lavender background. Branches are drawn to scale based on the number of CNA events. Chromosomal aberrations associated to the branch with the largest HG clone are shown. Red indicates gains and blue losses. Patient IDs highlighted in red indicate cases with coexisting PDAC. SNVs associated with each branch are labeled. Red arrow labels the branch with larger HG clone.

To model the evolutionary trajectory of CNAs, we applied the Copy-Number Tree Mixture Deconvolution (CNTMD). This method uses multiple samples of a tumor and aims to build evolutionary trees (35). We derived clonal evolution from CNAs by including events 5 Mb and higher. Using this approach, we detected a heterogeneous pattern compared with SNV evolution; only two of 23 (9%) HG lesions seem to follow a linear evolution by CNA analysis, while 10 of 23 (52%) were branched and11 of 23 (48%) could not be classified. In majority of cases (8/12), the evolutionary branch giving rise to the largest HG clone was associated with alterations in chromosome 1q (Fig. 3). When comparing clonal evolution derived from CNAs and SNVs, there was 67% (8/12) agreement. However, in 33% (4/12), the evolutionary trajectories were due to CNA-driven branching evolution. These results indicate that evolutionary trajectories solely based on SNVs could potentially miss CNA-driven subclonal evolution and thus underestimate a hidden branching lineage that may facilitate IPMN progression. We annotated our CNV-derived trees with the SNVs analyzed in Fig. 1B. Interestingly, in six of 10 cases with branched evolution by CNV, mutations were commonly shared between HG and LG lesions and were not specifically associated with the HG branch. For the remaining of the cases, in addition to *KRAS* and *GNAS*, mutations in *PIK3CA1*, *CTNNB1,* and *SLIT2* were identified in the lineage that gave rise to the HG (Fig. 3). Overall, our work reiterates the previously described heterogeneity that characterizes evolutionary trajectories of IPMN progression, which is further accentuated with the consideration of CNAs.

To integrate MACHINA and CNTNB analysis and gain a better understanding of the evolutionary trajectory associated with IPMN progression, we analyzed three cases in detail. Patient 9 LG and HG lesions shared an ancestral lineage defined by *KRAS^G12^, PIK3CA^E542K^*, and 66 additional SNVs. This clonal lineage gave rise to a dominant clone which contains eight additional SNVs, present at 2% in the LG, expanding to become a major clone in the HG (Supplementary Fig. S3A). In agreement, the evolutionary trajectory derived from CNAs indicated a linear evolution for HG which was marked by an amplification in chromosome 7 (Supplementary Fig. S3B and S3C). In patient 21, LG and HG lesions shared a common ancestor carrying *KRAS^G12R^* and *GNAS^R201C^* mutations. The evolutionary phylogeny derived from SNVs indicates linear evolution of LG to HG where in the latter, 14 additional mutations are acquired (Supplementary Fig. S3D). In contrast, the inferred clonal evolution derived from CNAs show evidence of CNA-driven branching with two clones present in the HG lesions. A major clone, unique for the HG lesion, showed gains in chromosomes 1, 3, 6, and 7 and losses in chromosomes 6, 11, and 22 which was confirmed by segmentation analysis (Supplementary Fig. S3D–S3F). In addition, a second clone shared between the LG and HG was exclusively characterized by chromosome 7 amplification. For patient 18, the SNV data showed clones in LG, HG, PDAC as having a most recent common ancestor clone with truncal *KRAS^G12V^* and *GNAS^R201C^* mutations. While there is a subclonal population unique to the LG and HG, the PDAC evolved independently from the dominant clone present in HG and LG (Supplementary Fig. S3G). Instead, a minor subpopulation, present at 3% in the HG, later expanded giving rise to the dominant PDAC clone defined by an *MTA1* mutation. Similarly, CNA-derived evolution showed a linear evolution from LG to HG which was characterized by gain in chromosome 1 and losses on chromosomes 6, 8, 18 as it was later clearly confirmed by chromosomal segmentation analysis. Of note, although both HG and tumor showed loss on chromosome 6, upon close inspection these losses occurred on different chromosomes, that is, one lineage lost the maternal copy while the other lost the paternal copy. Moreover, two additional branches were detected, the first branch was shared between the LG and PDAC while in the second branch, a completely independent PDAC clone, was identified with losses on chromosomes 1, 6, 9, 10, 16, and 18 (Supplementary Fig. S3H and S3I).

## Discussion

In this multi-institutional study, we interrogated the evolutionary trajectories and transcriptomic aberrations that occur during IPMN progression, using paired whole-exome DNA sequencing and whole-transcriptome sequencing. The majority of IPMNs within our dataset harbor somatic “hotspot” mutations in *KRAS* and *GNAS* as an early event, consistent with previously reported findings (36). In contrast to the prior study by Fischer and colleagues that found striking heterogeneity in driver gene mutations in IPMNs (26), we found driver SNVs to be relatively homogeneous, which may be the consequence of our more limited multiregion sequencing. On the contrary, our study suggests CNA events play a more pervasive role in the IPMN progression models than previously appreciated, such that accumulation of CNA events in a subgroup of LG IPMN seems to pave the way to further progression. In particular, we showed that HG samples with co-occurring PDAC tend to frequently harbor chromosomal 1q amplifications. Although it is important to underscore the limited number of patients in this cohort in the context of this potential stratifier, chromosome 1q amplification has also been detected in precancer lesions in other cancer types such as breast (37) and esophageal (38). In the context of PDAC, chromosome 1q amplifications have been previously described through SNP arrays and microarray-based comparative genomic hybridization, as well as most recently in the metastatic setting, but their relevance in noninvasive precursors is relatively unknown (39–41). The amplified region of chromosome 1q in IPMN harbors *PARP1,* whose product is an enzyme pivotal to DNA damage repair, including homologous recombination and error-prone DNA repair processes. An increase in PARP-1 enzymatic activity has been associated with the highly error-prone DNA repair pathway known as microhomology-mediated end joining, which has been reported to increase chromosomal structural alterations and genomic instability (42, 43). In our data, amplification in chromosome 1q, with concomitant overexpression of *PARP1*, was also associated with an increase in AS, suggesting that this event might be a prelude to genomic instability–enhanced progression in IPMNs.

We also confirm previous findings that from an evolutionary standpoint, IPMN progression is quite heterogenous (6, 7, 26, 34). Evolutionary modeling of our datasets demonstrates that while both linear and branched trajectories are present, a majority of IPMNs with co-occurring invasive phenotypes follow a branched evolution. Previous data had supported this evolutionary track with multiregion analysis of SNVs (7), but we now demonstrate how CNA-based phylogeny (with the added integration of transcriptome data) can identify similar patterns even with more limited sequencing analyses. For example, in patient 18 we found that the HG and PDAC areas show two distinct parental chromosomal deletions in chromosome 6q, distinct alterations in chromosome arms 1p and 1q and a transcriptomic class switch on RNA-seq, all of which point toward an independent development of the invasive PDAC from LG evolving in parallel to a co-occurrent HG lesion. Unfortunately, our results also indicate the challenges of predicting the pattern of progression in any given LG IPMN, given the inherent heterogeneity of possible pathways to HG and beyond.

Finally, it is worth noting that established transcriptomic PDAC signatures (20–22, 32) are also detectable in nearly all IPMN lesions. Signatures that have been previously suspected as likely originating from non-neoplastic tissue (exocrine-like and ADEX) were almost exclusively seen in ACs and normal ducts (21, 22), whereas LG, HG, and PDAC were mostly classified into the basal/squamous or classical/immunogenic subtypes. Consistent with our previous findings, this seems to demonstrate that even premalignant lesions express both consensus PDAC signatures and that certain pathways attributed to invasive carcinomas are even present within these lesions (44). Another interesting observation was that chromosome 1q amplifications were more common in samples expressing a classical subtype. Whereas patients with basal or basal-like subtypes are generally understood to experience poorer outcomes and response to systemic agents (45, 46), the abundance of a classical subtype and their association to 1q amplifications within this cohort and those profiled in TCGA may represent relevance within the preoneoplastic setting. In other words, classical subtypes may be more representative of preoneoplastic lesions during their stepwise trajectory to invasive disease, whereas basal subtypes are more of a hallmark of progression in an advanced setting which is supported by the finding that more IPMN with co-occuring PDAC harbor a basal subtype. It is also important to note the presence of several samples that were deemed to be nonclassifiable. This may be explained by the degraded nature of the RNA coming from FFPE tissues resulting in dropouts of classifier critical transcripts. Or perhaps these samples may represent a unique hybrid subtype as described previously (46). The transcriptomic data also revealed that reduction in transcripts associated with MHC class I antigen presentation machinery may be a feature of HG IPMNs. Recent work has shown reduced expression of MHC-I at the cell surface of PDAC cells which are targeted for lysosomal degradation (47, 48). Our data further suggest that perturbation of antigen presentation might occur even in noninvasive precursor lesions, and that there are other mechanisms beyond protein recycling to the lysosomes that may contribute to this dysfunction. Recent immune profiling data of IPMN progression have shown that the PME of noninvasive IPMNs is altered toward a more immune suppressive milieu upon progression to HG, and remarkably, comparable immune alterations are also observed in the matched LG IPMNs prior to progression (49). Interestingly, pathways relating to glycosylation were downregulated in HG compared with LG lesions, with previous work demonstrating the importance of orchestrated glycosylation of key proteins involved in antigen recognition and presentation to MHCI, with implications in immune response (50, 51). Although it is important to underscore the paucity of transcriptomic PME data within the current series, these findings suggest that the PME plays an integral permissive role in IPMN progression, with likely multiple mechanisms through which effective antigen presentation is perturbed early in multistep neoplasia.

## Supplementary Material

Supplementary Table 1 List of compartments dissected by LCM

Supplementary Table 2 Comparison in MAF and sequencing depth between a targeted and WES approach.

Supplementary Table 3 Correlation between PDAC molecular suptypes and 1q amplification in TCGA data

Supplementary Figure 1 Schematic representation of the strategy employed

Supplementary Figure 2 Comparison of SNVs, CNAs and neoepitopes in LG and HG IPMNs

Supplementary Figure 3 Evolutionary trajectory of IMPN lesions
