# Supplementary figures and images for: Integrated Molecular Characterization of Intraductal Papillary Mucinous Neoplasms: An NCI Cancer Moonshot Precancer Atlas Pilot Project
Source: Cancer Res Commun. 2023 Oct 10;3(10):2062–73. doi: 10.1158/2767-9764.CRC-22-0419 (PMC10563795; doi:10.1158/2767-9764.CRC-22-0419)

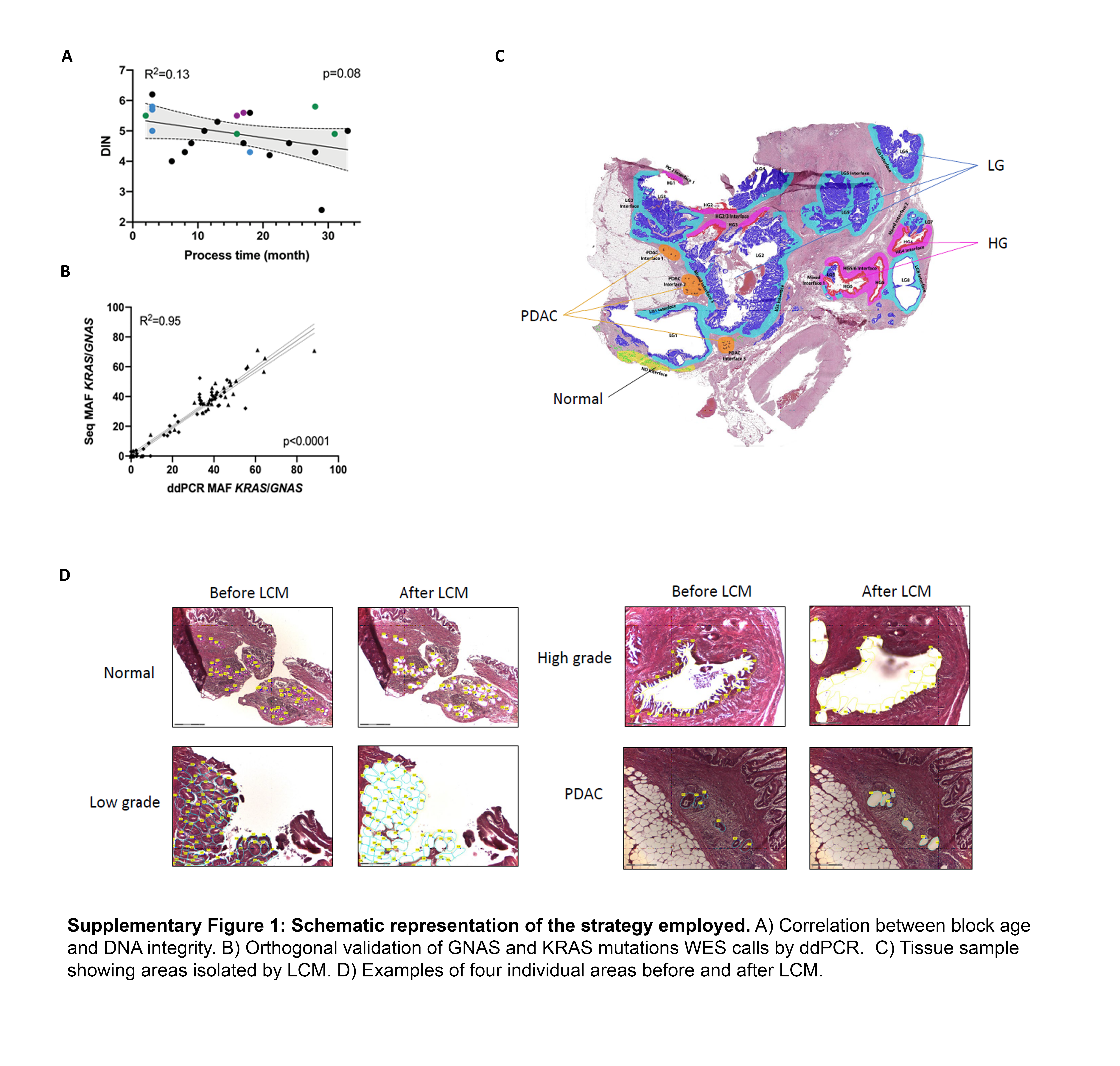

Supplement: Supplementary Figure 1 — Schematic representation of the strategy employed [file crc-22-0419-s04.png]

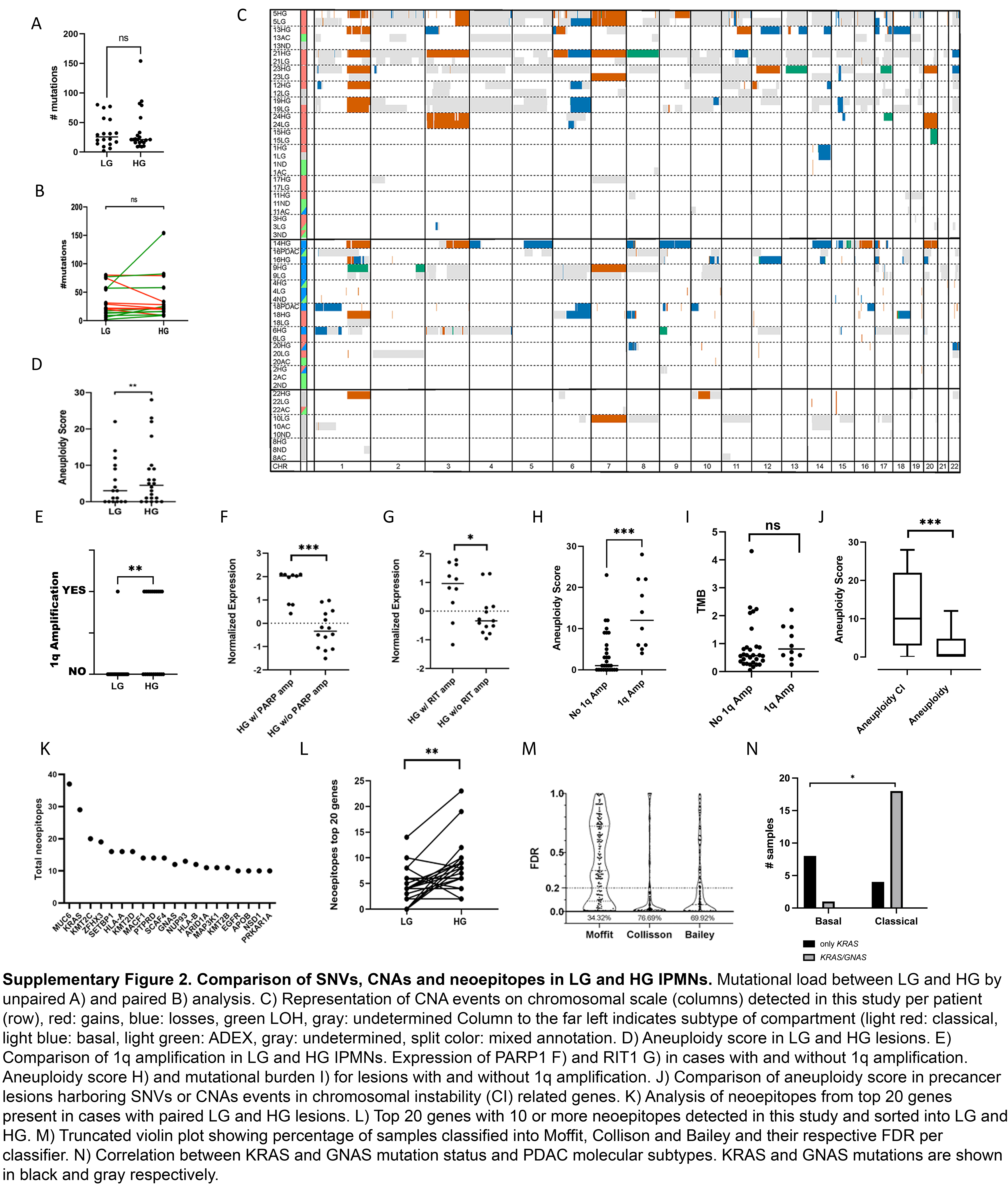

Supplement: Supplementary Figure 2 — Comparison of SNVs, CNAs and neoepitopes in LG and HG IPMNs [file crc-22-0419-s05.png]

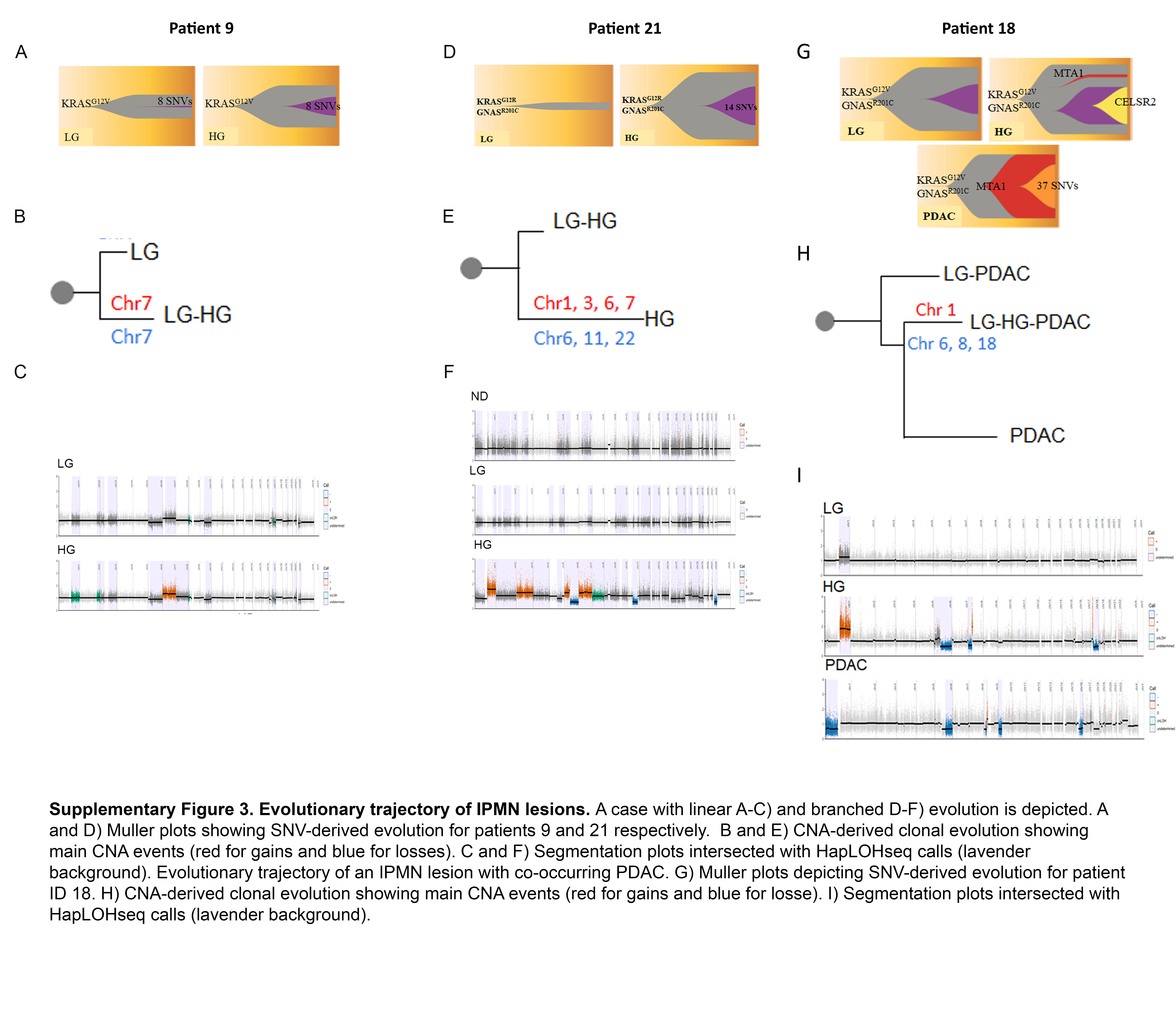

Supplement: Supplementary Figure 3 — Evolutionary trajectory of IMPN lesions [file crc-22-0419-s06.png]
